# Didymin protects against polystyrene nanoplastic-induced hepatic damage in male albino rats by modulation of Nrf-2/Keap-1 pathway

**DOI:** 10.1590/1414-431X2023e13173

**Published:** 2024-01-22

**Authors:** M.U. Ijaz, N. Nadeem, A. Hamza, M.H. Almutairi, U. Atique

**Affiliations:** 1 Department of Zoology, Wildlife and Fisheries, University of Agriculture, Faisalabad, Pakistan; 2 Department of Zoology, College of Science, King Saud University, Riyadh, Saudi Arabia; 3 College of Biological Systems, Chungnam National University, Daejeon, South Korea

**Keywords:** Polystyrene nanoplastics, Didymin, Antioxidant, Liver damage, Inflammation, Apoptosis

## Abstract

Polystyrene nanoplastics (PS-NPs) are ubiquitous environmental pollutants that can cause oxidative stress in various organs, including the liver. Didymin is a dietary flavanone that displays multiple pharmacological activities. Therefore, the present study evaluated the palliative role of didymin against PS-NPs-induced hepatic damage in rats. Albino rats (n=48) were randomly distributed into 4 groups: control, PS-NPs treated group, PS-NPs + didymin co-administered group, and didymin supplemented group. After 30 days, PS-NPs intoxication lowered the expression of *Nrf-2* and anti-oxidant genes [catalase *(CAT),* superoxide dismutase *(SOD),* glutathione peroxidase *(GPx),* glutathione reductase *(GSR),* glutathione-S-transferase *(GST),* and heme oxygenase-1 *(HO-1*)], whereas the expression of KEAP1 kelch like ECH associated protein 1 (Keap-1) was increased. PS-NPs exposure also reduced the activities of anti-oxidants enzymes (CAT, SOD, GPx, GSR, GST, GSH, and OH-1), while malondialdehyde (MDA) and reactive oxygen species (ROS) levels were increased. The levels of alanine transaminase (ALT), aspartate aminotransferase (AST), and alkaline phosphatase (ALP) were increased in PS-NPs-exposed rats. Moreover, inflammatory indices [interleukin-1β (IL-1β), tumor necrosis factor alpha (TNF-α), interleukin-6 (IL-6), nuclear factor-kappa B (NF-κB), and cyclooxygenase-2 (COX-2)] were increased in PS-NPs-exposed rats. Furthermore, PS-NPs intoxication increased the expressions of apoptotic markers including *Bax* and *Caspase-3*, as well as reducing *Bcl-2* expression. The histopathological analysis showed significant damage in PS-NPs-treated rats. However, didymin supplementation ameliorated all the PS-NPs-induced damage in the liver of rats. Therefore, it was concluded that didymin can act as a remedy against PS-NPs-induced liver toxicity due to its anti-apoptotic, anti-oxidant, and anti-inflammatory activities.

## Introduction

Numerous environmental toxins can affect the organs of the body. Special focus is being devoted to emerging contaminants, such as plastic waste, plasticizers, and plastic additives, which are released into the environment directly or indirectly (1). It is evident that plastic pollution is so pervasive in the environment that we can say that our world is made of plastic (2). In 2019, global plastic production reached 368 million metric tons (Mt) and is expected to double in the next twenty years (3). Polystyrene (PS) is an important thermostable plastic that is often used in the manufacturing of toys, CDs, electronics, toothbrushes, packaging foam, and several other personal care products (4).

Nanoplastics are plastic fragments with a diameter of around 1000 nm that are formed from larger plastics by photodegradation, chemical deterioration, and wave erosion (5). Polystyrene nanoplastics (PS-NPs) are new environmental pollutants that have been detected in the air, drinking water, and human foods such as seafood, tea bags, beer, table salt, vegetables, honey, fruits, and sugar (6). Human exposure to PS-NPs occurs through multiple ways such as inhalation, ingestion, and dermal contact (7). PS-NPs can easily cross biological membranes due to their small size and, thus, accumulate in tissues and subsequently induce physiological damages (8). PS-NPs exposure induces reactive oxygen species (ROS) production that leads to endoplasmic reticulum and oxidative stress (9,10). The liver is a metabolic center that plays an important role in the majority of metabolic illnesses, such as diabetes and obesity. PS-NPs have the ability to induce hepatic inflammation, alterations in the lipid profile, and cholesterol buildup in the liver (11).

Flavonoids are a class of secondary metabolites that are abundant in many plants. Due to their proven effects in the prevention and treatment of multiple ailments, flavonoids have gained the attention of researchers (12). Didymin is a dietary glycoside flavanone that is reported in campanula and citrus fruits such as mandarin, orange, bergamot, and oregano. Recent studies have demonstrated that didymin has cardio-protective, anti-oxidant, and anti-cancer potentials (13). Based on these putative pharmacological properties of didymin, the current study was designed to determine the protective role of didymin on PS-NPs-induced hepatic damage.

## Material and Methods

### Chemicals

PS-NPs and didymin were purchased from Sigma-Aldrich, Germany.

### Animals

Albino rats (n=48) weighing 180±20 g (6-8 weeks old) were used for this research. The animals were acquired from the Animal House of the University of Agriculture, Faisalabad (UAF), and kept in stainless-steel cages at 24-26°C with a 12-h light/dark cycle. During the whole trial, rats were given free access to water and commercial feed. Rats were treated and handled according to the protocol of European Union of Animal Care and Experimentation (CEE Council 86/609) that was further approved by University of Agriculture, Faisalabad, Ethical Committee (20189-92/01-06-2023).

### Experimental layout

The 48 albino rats were allocated into 4 groups of twelve rats each: control, PS-NPs (50 µg/kg) exposure, PS-NPs (50 µg/kg) + didymin (1 mg/kg), and didymin (1 mg/kg) supplementation. All substances were given through oral gavage. After 30 days of treatment, the rats were anesthetized with 6 mg/kg of xylazine and 60 mg/kg of ketamine (Sigma Aldrich, Germany) (14,15) and were decapitated; cardiac blood was collected in heparinized tubes. Serum samples were obtained by centrifuging the blood at 1000 *g* for 15 min at room temperature and stored at -20°C for biochemical assays. The liver was removed and sliced into two equal parts. One part was packed in zip bags and stored at -80°C for biochemical analysis, while the other part was fixed in 10% formalin for histopathological examination.

### Evaluation of antioxidant enzymes

Catalase (CAT) activity was estimated following the procedure of Aebi (16). Superoxide dismutase (SOD) activity was assessed using the protocol of Kakkar et al. (17), and glutathione peroxidase (GPx) activity was determined using the procedure elucidated by Rotruck et al. (18). Glutathione reductase (GSR) activity was determined by following the technique of Carlberg and Mannervik (19), and the activity of glutathione-S-transferase (GST) was evaluated using the protocol of Habig et al. (20). Glutathione (GSH) activity in hepatic tissue was appraised using the method of Jollow et al. (21), and heme oxygenase-1 (HO-1) activity was determined by analyzing the formation of bilirubin using the method of Magee et al. (22).

### Evaluation of oxidative stress markers

Malondialdehyde (MDA) and ROS levels were evaluated using the techniques of Ohkawa et al. (23) and Hayashi et al. (24), respectively.

### Ribonucleic acid extraction and real-time quantitative reverse transcription polymerase chain reaction

The expression of *Nrf-2/Keap-1,* anti-oxidant genes, and *Caspase-3, Bcl-2, and Bax* were determined by qRT-PCR. RNA was separated using TRIzol reagent (Sigma Aldrich). Total RNA was changed into complementary DNA with Fast Quant RT kit (Takara, China). Changes in these expressions were appraised by 2^-ΔΔCT^ using β-actin as an internal control. Table 1 displays the primer sequences of the target genes, as reported previously (25,26).

**Table 1:** Primer sequences for the real-time quantitative reverse transcription-polymerase (RT-qPCR).

Gene	Primers 5'->3'	Accession number
*Nrf-2*	F: ACCTTGAACACAGATTTCGGTG	NM_031789.1
	R: TGTGTTCAGTGAAATGCCGGA	
*Keap-1*	F: ACCGAACCTTCAGTTACACACT	NM_057152.1
	R: ACCACTTTGTGGGCCATGAA	
*CAT*	F: TGCAGATGTGAAGCGCTTCAA	NM_012520.2
	R: TGGGAGTTGTACTGGTCCAGAA	
*SOD*	F: AGGAGAAACTGACAGCTGTGTCT	NM_017051.2
	R: AAGATAGTAAGCGTGCTCCCAC	
*GPx*	F: TGCTCATTGAGAATGTCGCGTC	NM_030826.4
	R: ACCATTCACCTCGCACTTCTCA	
*GSR*	F: ACCAAGTCCCACATCGAAGTC	NM_053906.2
	R: ATCACTGGTTATCCCCAGGCT	
*GST*	F: TCGACATGTATGCAGAAGGAGT	NM_031509.2
	R: CTAGGTAAACATCAGCCCTGCT	
*HO-1*	F: AGGCTTTAAGCTGGTGATGGC	NM_012580.2
	R: ACGCTTTACGTAGTGCTGTGT	
*Bax*	F: GGCCTTTTTGCTACAGGGTT	NM_017059.2
	R: AGCTCCATGTTGTTGTCCAG	
*Bcl-2*	F: ACAACATCGCTCTGTGGAT	NM_016993.1
	R: TCAGAGACAGCCAGGAGAA	
*Caspase-3*	F: ATCCATGGAAGCAAGTCGAT	NM_012922.2
	R: CCTTTTGCTGTGATCTTCCT	
*β-actin*	F: TACAGCTTCACCACCACAGC	NM_031144
	R: GGAACCGCTCATTGCCGATA	

F: forward; R: reverse.

### Hepatic function markers

The level of liver function enzymes, i.e., aspartate aminotransferase (AST), alkaline phosphatase (ALP) and alanine transaminase (ALT), was assessed using commercial kits from Wiesbaden (Germany).

### Analysis of inflammatory indices

Inflammatory indices, i.e., tumor necrosis factor alpha (TNF-α), nuclear factor-kappa B (NF-kβ), interleukin (IL)-1β, and IL-6 levels and cyclooxygenase-2 (COX-2) activity, were estimated using the ELISA kits according to manufacturer’s instructions (BioTek, USA).

### Histopathological analysis

For histopathological observation, hepatic tissues were gently washed with cold saline solution. Later, hepatic tissues were fixed in 10% formalin solution. Then, the tissues were gradually dehydrated by passing through rising grades (70, 90, and 100%) of ethanol and embedding was performed in paraffin wax. Five micrometer-thick sections were made with a 820 Spencer rotary microtome (Indiamart, Ambala, Haryana) and stained by using hematoxylin and eosin. Finally, the slides were examined under a light microscope that was equipped with an automatic photographic system (Nikon, Japan).

### Statistical analysis

Data are reported as means±SEM. One-way ANOVA and Tukey's test were applied using Minitab software (USA). The level of significance was set at P<0.05.

## Results

### Effect of didymin on Nrf-2/Keap-1 pathway

**Figure 1:**
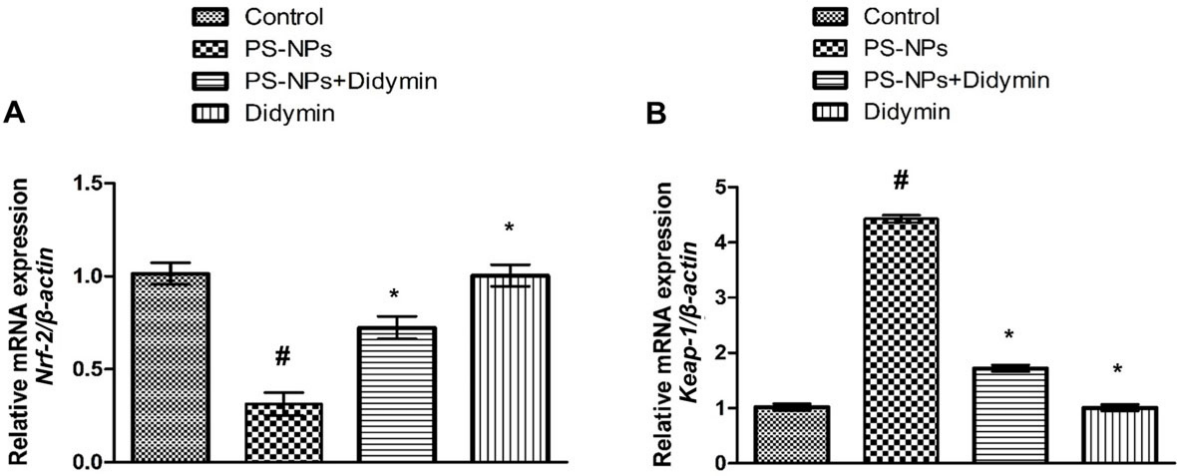
Effect of polystyrene nanoplastics (PS-NPs) and didymin on the expression of **A**, *Nrf-2* and **B**, *Keap-1*. Data are reported as means±SEM. ^#^P<0.05 compared to control, *P<0.05 compared to PS-NPs-treated group (ANOVA).

**Figure 2:**
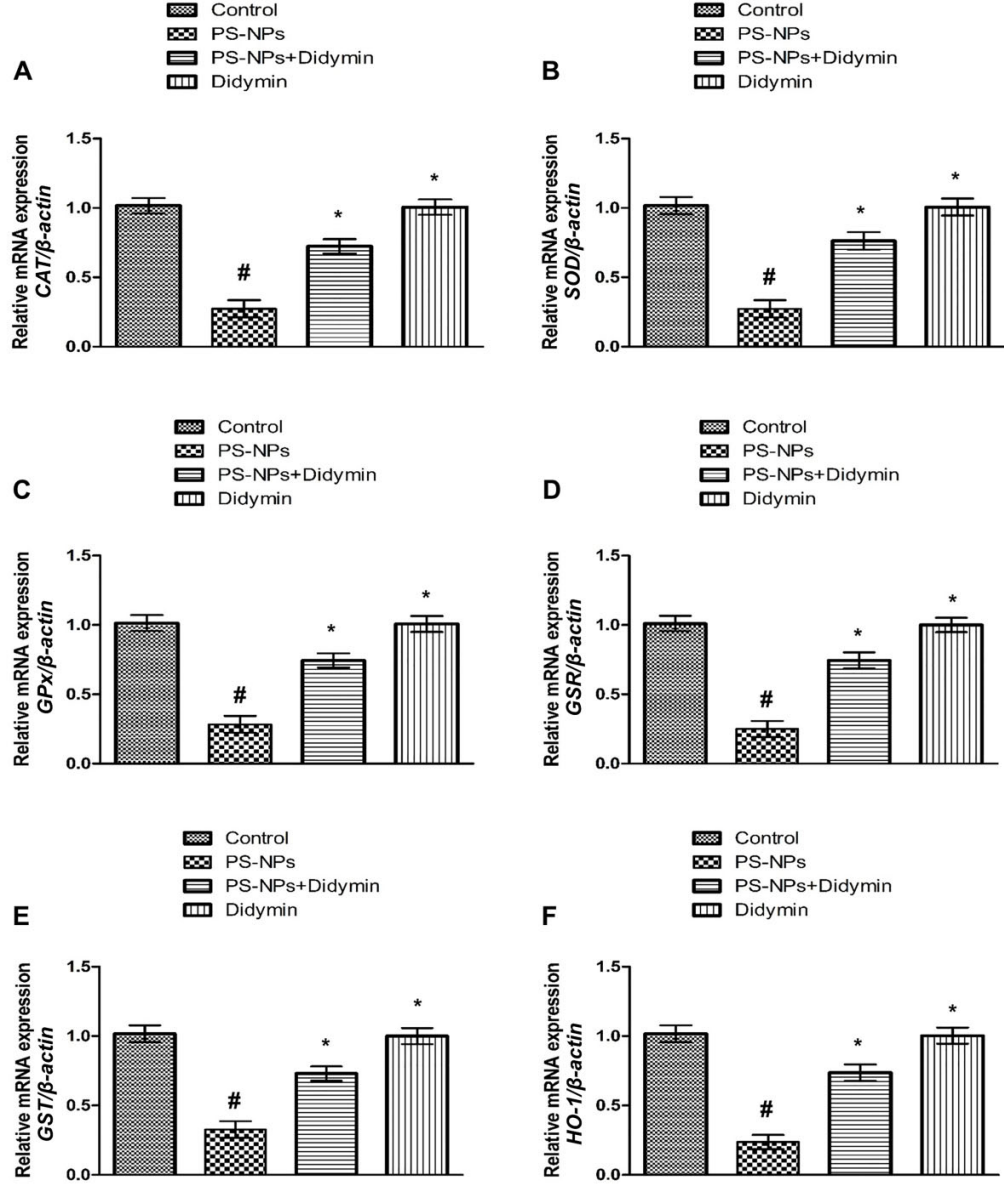
Effect of polystyrene nanoplastics (PS-NPs) and didymin on the expression of **A**, catalase (*CAT*); **B**, superoxide dismutase (*SOD*); **C**, glutathione peroxidase (*GPx*); **D**, glutathione reductase (*GSR*); **E**, glutathione-S-transferase (*GST*); and **F**, heme oxygenase-1 (*HO-1*). Data are reported as means±SEM. ^#^P<0.05 compared to control, *P<0.05 compared to PS-NPs-treated group (ANOVA).

PS-NPs exposure prompted a significant (P<0.05) decrease in the expressions of *Nrf-2* and anti-oxidant genes (*SOD*, *GPx*, *CAT*, *GST*, *HO-1*, and *GSR*), whereas the expression of *Keap-1* in PS-NPs-exposed rats was increased compared to the control animals. The administration of PS-NPs with didymin increased *Nrf-2* and anti-oxidant enzyme expressions, as well as down-regulating *Keap-1* expression. Moreover, the results of didymin-only supplemented rats were comparable to the control rats (Figures 1 and 2).

### Effect of didymin on anti-oxidant activity

Intoxication by PS-NPs resulted in a significant (P<0.05) reduction in the activities of anti-oxidants SOD, CAT, GSR, GPx, GST, HO-1, and GSH in the PS-NPs-exposed group in contrast to the control animals. In the PS-NPs + didymin co-treated group, the activities of anti-oxidant enzymes were significantly increased compared to the PS-NPs group. Furthermore, the anti-oxidant activities in didymin-only group were similar to that of the control group (Table 2).

**Table 2:** Effect of polystyrene nanoplastics (PS-NPs) and didymin on biochemical parameters.

Parameters	Groups			
	Control	PS-NPs	PS-NPs+Didymin	Didymin
CAT (mU/g protein)	9.64±0.55	4.55±0.32^#^	7.85±0.25*	9.67±0.54*
SOD (Um/g protein)	7.72±0.23	2.86±0.33^#^	5.97±0.29*	7.75±0.23*
GPx (Um/g protein)	26.46±1.14	6.63±0.46^#^	17.74±1.08*	26.63±1.20*
GSR (nM NADPH oxidized/min per mg tissue)	6.31±0.22	1.94±0.19^#^	5.19±0.29*	6.33±0.21*
GST (nM/min per mg protein)	32.85±1.18	12.19±1.40^#^	26.45±0.68*	23.31±17.87*
GSH (μM/g tissue)	19.08±0.50	3.85±0.26^#^	16.72±0.68*	19.18±0.55*
HO-1 (pmoles bilirubin/mg protein per h)	235.48±10.56	42.70±6.48^#^	155.93±11.99*	234.40±13.39*
ROS (Um/g tissue)	1.33±0.09	8.19±0.33^#^	2.29±0.22*	1.31±0.09*
MDA (nmol/mg protein)	0.79±0.12	6.84±0.24^#^	1.33±0.21*	0.77±0.13*

Data are reported as means±SEM. ^#^P<0.05 compared to control, *P<0.05 compared to PS-NPs-treated group (ANOVA). CAT: catalase; SOD: superoxide dismutase; GPx: glutathione peroxidase; GSR: glutathione reductase; GST: glutathione-S-transferase; HO-1: heme oxygenase-1; MDA: malondialdehyde; ROS: reactive oxygen species.

### Effect of didymin on oxidative stress markers

PS-NPs significantly (P<0.05) increased the levels of MDA and ROS in contrast to the control animals. However, the PS-NPs + didymin group had significantly lower MDA and ROS levels compared to PS-NPs rats. Furthermore, these levels were almost similar to the didymin-only and control groups (Table 2).

### Effect of didymin on hepatic function markers

PS-NPs significantly (P<0.05) increased the levels of AST, ALT, and ALP compared to the control rats. However, PS-NPs + didymin co-treatment led to a substantial decrease in the levels of AST, ALT, and ALP compared to the PS-NPs group. Furthermore, in didymin-only treated group, these levels were similar to the control group (Table 3).

**Table 3:** Effect of polystyrene nanoplastics (PS-NPs) and didymin on liver function markers.

Parameters	Groups			
	Control	PS-NPs	PS-NPs + Didymin	Didymin
ALT (U/L)	42.83±1.71	88.16±3.61^#^	58.28±2.79*	42.81±1.71*
AST (U/L)	76.54±2.69	187.07±4.52^#^	93.08±2.31*	76.51±2.69*
ALP (U/L)	125.55±2.15	348.57±7.18^#^	190.41±5.60*	124.64±3.10*

Data are reported as means±SEM. ^#^P<0.05 compared to control, *P<0.05 compared to PS-NPs-treated group (ANOVA). ALT: alanine transaminase; AST: aspartate aminotransferase; ALP: alkaline phosphatase.

### Effect of didymin on apoptotic markers

**Figure 3:**
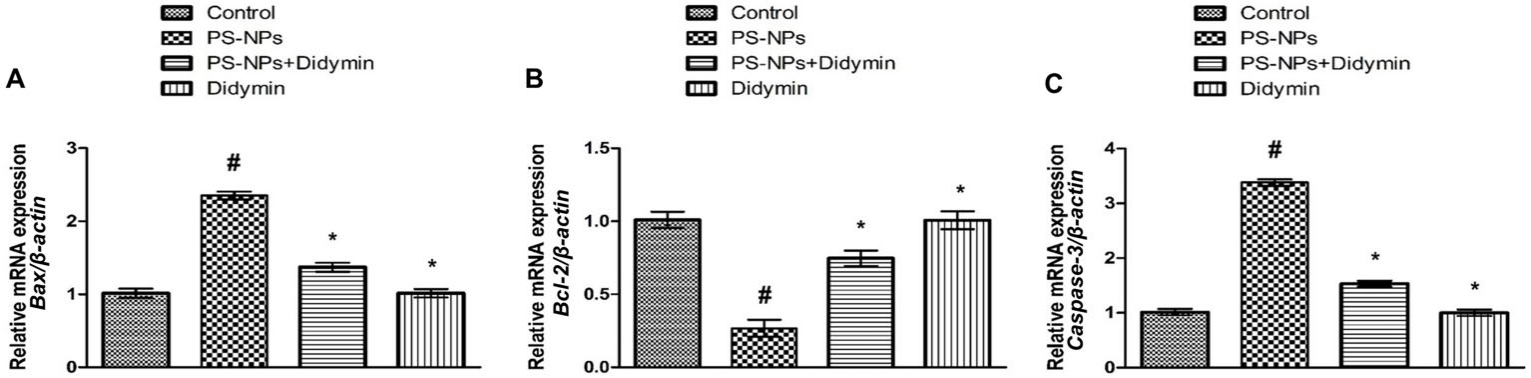
Effect of polystyrene nanoplastics (PS-NPs) and didymin on the expression of **A**, *Bax*; **B**, *Bcl-2*; and **C**, *Caspase-3*. Data are reported as means±SEM. ^#^P<0.05 compared to control, *P<0.05 compared to PS-NPs-treated group (ANOVA).

The exposure of PS-NPs significantly (P<0.05) increased the expressions of *Caspase-3* and *Bax*, as well as reducing the *Bcl-2* expression compared to the control group. Nevertheless, supplementation of PS-NPs + didymin significantly reduced Caspase-3 and Bax expression, while increasing the Bcl-2 expression in contrast to the control group. Moreover, no significant change was observed in the expressions of these markers in didymin-only and control groups (Figure 3).

### Effect of didymin on inflammatory markers

Inflammatory indices of IL-6, NF-κB, IL-1β, and TNF-α levels and COX-2 activity in the liver of the PS-NPs rats were significantly (P<0.05) elevated in contrast to control rats. However, compared to the PS-NPs group, these markers were notably decreased in the co-treated (PS-NPs + didymin) group. Moreover, no significant variations were observed in inflammatory indices between didymin-alone and control groups (Table 4).

**Table 4:** Effect of polystyrene nanoplastics (PS-NPs) and didymin on liver inflammatory markers.

Parameters	Groups			
	Control	PS-NPs	PS-NPs + Didymin	Didymin
NF-κB (ng/g tissue)	12.73±1.01	83.28±2.01^#^	21.77±1.17*	12.65±1.00*
TNF-α (ng/g tissue)	7.26±0.56	47.03±1.77^#^	17.20±1.26*	7.22±0.54*
IL-1β (ng/g tissue)	23.06±1.52	89.58±2.07^#^	37.32±2.20*	22.95±1.51*
IL-6 (ng/g tissue)	10.43±0.82	65.53±1.86^#^	25.35±2.16*	10.41±0.72*
COX-2 (ng/g tissue)	14.87±1.27	85.90±1.43^#^	34.34±1.34*	14.85±1.28*

Data are reported as means±SEM. ^#^P<0.05 compared to control, *P<0.05 compared to PS-NPs-treated group (ANOVA). NF-κB: nuclear factor-kappa B; TNF: tumor necrosis factor; IL: interleukin; COX: cyclooxygenase.

### Effect of didymin on liver histology

**Figure 4:**
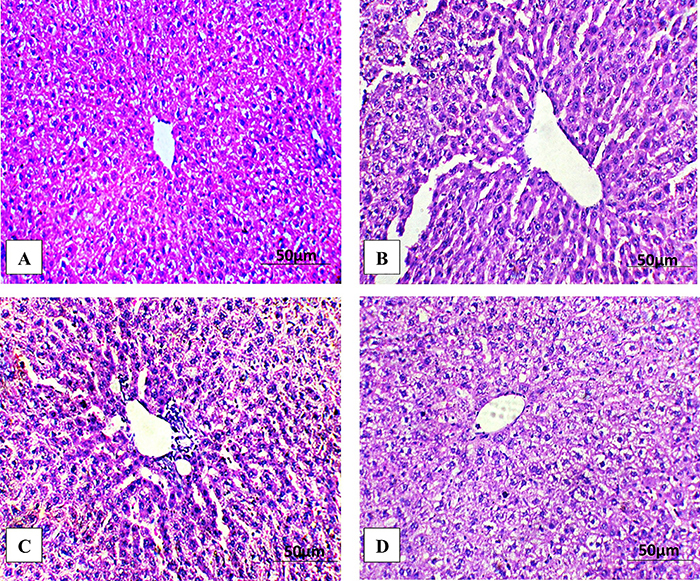
Photomicrographs of rat hepatic tissues. Hematoxylin and eosin staining; scale bar 50 μm. **A**, Control group presenting normal histology. **B**, Polystyrene nanoplastics (PS-NPs) intoxication prompted adverse alterations in the liver. **C**, PS-NPs + didymin group displayed improved histology of liver tissues. **D**, Didymin group showing normal histology similar to the control rats.

PS-NPs intoxication resulted in histopathological damages, such as degenerated hepatocytes, nuclear dissolution in necrotic cells, degeneration of lobules and nucleus, congested central veins, and dilated sinusoid. However, the supplementation of PS-NPs + didymin significantly decreased these damages compared to the PS-NPs-exposed group. Moreover, didymin-alone supplemented rats showed normal liver histology comparable to the control rats (Figure 4).

## Discussion

Exposure to PS-NPs decreased the expressions of *Nrf-2* and anti-oxidant genes (*CAT*, *SOD*, *GPx*, *GSR*, and *OH-1*), whereas the expression of *Keap-1* increased. *Nrf-2* is an important transcription factor that has a central role in oxidative and electrophilic stress control. In addition, Keap-1 interacts with Nrf-2, regulating its stability and acting as its inhibitor (27). During ROS production, *Nrf-2* detaches from Keap-1 through some structural modifications and migrates to the nucleus where it interacts with small MAF proteins. Then, the heterodimers bind to the anti-oxidant responsive elements and activate the expression of cytoprotective genes (28). *Nrf-2* plays a pivotal role in controlling the expression of anti-oxidant enzymes (CAT, SOD, GPx, GSR, and OH-1) (29). However, under excessive ROS production, the expression of *Keap-1* increases while the expression of *Nrf-2* decreases (30). Therefore, decreased *Nrf-2* expression reduces the expression of antioxidant genes. However, didymin supplementation increased the expression of *Nrf-2* that was further confirmed by the elevated expression of anti-oxidant genes.

PS-NPs intoxication remarkably reduced the activities of CAT, SOD, GSR, GPx, GSH, HO-1, and GST, while the levels of MDA and ROS increased. The antioxidant defense system is exhausted when the amount of ROS produced surpasses the capacity of antioxidants (31). PS-NPs exposure not only reduces the activities of antioxidant enzymes but also induces lipid peroxidation and oxidative stress in the hepatic tissues of rats. The endogenous antioxidant enzymes SOD, GPx, and CAT are regarded as the first line of defense as they lower the oxidative stress (32). CAT promotes the breakdown of H_2_O_2_ to O_2_ and H_2_O by limiting the production of the hazardous ions OH^-^. SOD transforms superoxide free radicals into H_2_O_2_ and O_2_. To mitigate oxidative stress, GPx reduces H_2_O_2_ and lipid peroxide levels. GST plays an important part in the detoxification process in liver tissues by promoting the bonding of GSH to xenobiotic substrates. HO-1 is a cytoprotective enzyme with the ability to breakdown the heme and plays a notable role in the regulation of cellular homeostasis. MDA is an indicator of lipid peroxidation and its level is directly related to the level of lipid peroxidation. In this study, didymin administration significantly increased anti-oxidant activities and decreased the levels of MDA and ROS due to its anti-oxidant and radical scavenging property.

PS-NPs intoxication significantly increased the serum levels of ALT, ALP, and AST. The evaluation of these enzymes in the blood is one of the most widely used methods for analyzing hepatic damage. Hepatocyte apoptosis causes the liver mitochondria to release these enzymes into the bloodstream, resulting in liver dysbiosis. According to earlier studies, the excessive production of ROS affects the integrity of the liver, as indicated by the unusual increase in the level of hepatic serum enzymes (33). However, didymin reduced the levels of these hepatic enzymes due to its hepatoprotective nature.

Apoptosis is one of the major causes of hepatic damage. Bcl-2 and Bax are associated with the Bcl-2 protein family, which controls the mitochondrial apoptotic pathway. Bax is an apoptotic marker whereas Bcl-2 defends the cells from apoptosis. An elevation in *Bax* expression and a decrease in *Bcl-2* expression prompts the release of cytochrome-C into the cytoplasmic matrix from the mitochondrial membrane, which activates Caspase-3. Caspase-3 is a member of the cysteine protease family, which is involved in the breakdown of cellular proteins and leads to apoptotic cell death (34). In our study, didymin supplementation decreased *Caspase-3* and *Bax* expressions, while *Bcl-2* expression was increased due to its anti-apoptotic nature.

PS-NPs administration in rats led to a significant increase in inflammatory indices (IL-1β, NF-kB, IL-6, TNF-α, and COX-2). Oxidative stress stimulates NF-kB, which activates the transcription of several inflammatory markers (IL-1β, TNF-α, and IL-6), which eventually leads to hepatic damage. COX-2 is also an inflammatory marker that has a pivotal role in the inflammation process and tissue damage (35). Didymin supplementation remarkably reduced the levels of inflammatory markers, which might be attributed to its anti-inflammatory property.

The histomorphological analysis of hepatic tissues revealed that PS-NPs exposure caused various damages. The oxidative damage caused by free radicals can potentially damage macromolecules, which leads to cellular damage and degeneration of hepatic tissues as well as necrosis (36). However, didymin administration efficiently reduced all the PS-NPs-induced histopathological damages in the liver of rats, which may be attributed to its anti-apoptotic, anti-oxidant, and anti-inflammatory potentials.

### Conclusion

In conclusion, didymin seemed to be protective against liver damage from PS-NPs.
